# Metformin suppressed the proliferation of LoVo cells and induced a time-dependent metabolic and transcriptional alteration

**DOI:** 10.1038/srep17423

**Published:** 2015-11-30

**Authors:** Jiaojiao He, Ke Wang, Ningning Zheng, Yunping Qiu, Guoxiang Xie, Mingming Su, Wei Jia, Houkai Li

**Affiliations:** 1 Center for Chinese Medical Therapy and Systems Biology, Shanghai University of Traditional Chinese Medicine, Shanghai 201203, China; 2 School of Public Health, Shanghai University of Traditional Chinese Medicine, Shanghai 201203, China; 3 Laboratory of Integrative Medicine Surgery, Shuguang Hospital, Shanghai University of Traditional Chinese Medicine, Shanghai 201203, China; 4 Center for Translational Medicine, and Shanghai Key Laboratory of Diabetes Mellitus, Department of Endocrinology and Metabolism, Shanghai Jiao Tong University Affiliated Sixth People’s Hospital, Shanghai 200233, China; 5 Stable Isotope and Metabolomics Core Facility, Diabetes Center Albert Einstein College of Medicine, 1300 Morris Part Ave, Bronx, New York, 10461, USA; 6 Cancer Epidemiology Program, University of Hawaii Cancer Center, Honolulu, Hawaii, 96813, USA

## Abstract

Metformin is a widely used anti-diabetic drug with potential anti-tumor activity. However, little is known about its global metabolic and transcriptional impacts on tumor cells. In current study, we performed a metabolic profiling on human-derived colon cancer LoVo cells treated by 10 mM metformin for 8, 24 and 48 h. An obvious time-dependent metabolic alteration was observed from 8 to 48 h, prior to the reduction of cell viability. A total of 47, 45 and 66 differential metabolites were identified between control and metformin-treated cells at three time points. Most of the metabolites were up-regulated at 8 h, but down-regulated at 24 and 48 h by metformin. These metabolites were mainly involved in carbohydrates, lipids, amino acids, vitamins and nucleotides metabolism pathways. Meanwhile, the transcirptomic profile revealed 134 and 3061 differentially expressed genes at 8 and 24 h by metformin. In addition to the cancer signaling pathways, expression of genes involved in cell energy metabolism pathways was significantly altered, which were further validated with genes in glucose metabolism pathway. Altogether, our current data indicate that metformin suppressed the proliferation of LoVo cells, which may be due to the modulation on cell energy metabolism at both metabolic and transcriptional levels in a time-dependent way.

Colorectal cancer is one of the leading causes of tumor-associated death worldwide, and a higher risk of colorectal cancer is observed in patients with type 2 diabetes^1^,^2^. The observational studies indicate that metformin treatment lowers the risk of colon cancer in type 2 diabetes patients^3^, and several lines of experimental evidence suggest that the mechanisms underlying the suppression on aberrant crypt foci formation of metformin are associated with the inhibition of mTOR resulted from the activation of AMPK^4^. However, very little is known about the global metabolic impacts of metformin linking to the colon cancer development.

The alteration in cell energy metabolism is a hallmark of tumor cells, which are more dependent on aerobic glycolysis to generate ATP for cell growth. Metformin is a potent activator of AMP-activated protein kinase (AMPK), which plays a crucial role in modulating cell energy metabolism and insulin sensitivity. The anti-tumor property of metformin is proposed in either AMPK-dependent or –independent way^5^,^6^. The molecular mechanism involves inhibition of mammalian target of rapamycin complex I (mTORC1)^7^, as well as the induction of p53-dependent cell cycle arrest and apoptosis^8^,^9^. In addition, metformin is also a poisoner of mitochondria by impairing the function of complex I^10^, leading to the increased aerobic glycolysis as compensation . The suppression of complex I prevents NADH oxidation, which results in the requirement for cytosolic NADH being oxidized by converting pyruvate to lactate. Given the fact of complicated metabolic impacts of metformin on either metabolic diseases or tumors, the omics-based approaches are powerful for deciphering the global effects of metformin on tumors.

Metabolomics holds the advantages of revealing the comprehensive metabolic alterations in a biological system either alone or in combination with other omics approaches. In breast cancer cells, metabolomic fingerprint indicates that metformin treatment results in significant accumulation of 5-formimino-tetrahydrofolate, and the supplementation of hypoxanthine for purine salvage pathway greatly attenuates the anti-tumor effect of metformin^11^. This metabolomic-based study uncovers that metformin can function as antifolate chemotherapeutic agent that induces tumor suppressor through the folate-related one-carbon metabolic pathways. Meanwhile, the global metabolic impacts of metformin have also been investigated in a Src-inducible model of cellular transformation and breast cancer stem cells^12^. The results show that metformin decreases the intermediates of glycolysis and TCA cycle, as well as depletion of nucleotide triphosphates, which are consistent with the well-established effect of metformin on inhibiting the activity of mitochondrial complex I.

In the present study, we performed a combined metabolomic and transcriptomic study on the global effects of metformin with different culture time on a human-derived colon cancer LoVo cells. Our results indicate metformin treatment exerts transparent impacts on LoVo cells both at transcriptional and metabolic levels earlier than the appearance of cell viability reduction. The metabolomic data indicate that most of the cellular metabolites are depleted during the culture period from 8 to 48 h in control LoVo cells, whereas metformin treatment accelerates the depletion of cellular metabolites at 24 and 48 h, except for at 8 h. Meanwhile, the transcriptomic results indicate that metformin treatment resulted in over 130 and 3000 differentially expressed genes at 8 and 24 h, respectively. The combined metabolic and transcriptional results suggest the cell energy metabolism pathway is the main target of metformin.

## Experimental Section

### Cell culture and treatment

Human-derived colon cancer LoVo cells (CCL-229) from ATCC were routinely cultured in 10 cm dishes at 37 °C in a humidified atmosphere of 5% CO_2_ in 10% FBS DMEM supplemented with 100 U/ml penicillin and 100 mg/ml streptomycin. The metabolic profiling was performed on cells treated with or without 10 mM metformin (Sigma Aldrich, USA) for 8, 24 and 48 h, respectively, while the transcriptomic profiling was conducted on cells after 8 and 24 h of metformin treatment.

### Determination of cell viability

Cells were seeded in 96-well plates at 2 × 10^4^ cells/well in 10% FBS DMEM (GIBCO, USA). After 24-h culture, cells were treated with Metformin at different concentrations from 0, 0.5, 1, 2.5, 5, and 10 mM in 10% FBS DMEM for 24 h. Then, cell viability was determined using the cell counting kit-8 according to the instructions.

### Metabolic profiling of cells

The cellular metabolic profiling was carried out on GC/TOFMS (Pegasus HT system, Leco Corporation) and LC/TOFMS (Agilent Corporation) after the two-step extraction of cellular metabolites with mixed solvent. The cellular metabolite extraction process was described as following: Place the 10 cm dishes containing about 1 × 10^7^ cells/each on ice. Remove the culture medium and wash with 5 ml of ice-cold isotonic saline (0.9% [w/v] NaCl for three times. Add 1 ml of ice-cold 0.9% NaCl and scrape the cells off carefully. Transfer the cells into a 1.5 ml centrifuge tube, and centrifuge at 1000 × g for 2 min at 4 °C. Remove the supernatant and repeat the washing step twice with 0.9% NaCl. After that, add 350 μl of methanol : chloroform : water (2.5:1:1 [v/v/v]) mixed solvent which has been refrigerated at −20 °C completely. Vortex for 30 s and sonicate for 5 min in ice water. Centrifuge at 13,200 rpm for 15 min at 4 °C. Transfer 175 μl of supernatant to a GC and LC sampling vial respectively for the following GC/TOFMS and LC/TOFMS analysis. The sample residue was extracted with 350 μl of methanol again. Vortex for 30 s, and sonicate for 5 min. Centrifuge at 13,200 rpm for 15 min at 4 °C, and transfer 175 μl of supernatant to the GC and LC sampling vials respectively. Then, each sample was added with internal standards (10 μL heptadecanoic acid at 1 mg/mL and 4-chlorophenylalanine at 0.3 mg/mL). For GC/TOFMS analysis, the metabolite extraction was vacuum dried at room temperature, and the residue was then chemically derivatized with BSTFA, while the LC/TOFMS was performed directly with the solvent extraction. The protocols for GC/TOFMS and LC/TOFMS were described previously^13^,^14^.

### Transcriptomic profiling of cells

After the time course experiments, cells were collected and total RNA was extracted using TRIzol reagent (Invitrogen, Carlsbad, CA), followed by purification on an RNeasy column (Qiagen, Germantown, MD) and quantified by UV absorption (Nanodrop, Thermo Scientific). RNA quality was evaluated by RNA 6000 Nano LabChip (Agilent Technologies, Santa Clara, CA) on an Agilent 2100 Bioanalyzer. Following quantification, 1 μg of each total RNA sample was amplified, labeled and hybridized according to the standard Affymetrix protocols by Expression Analysis, Inc. (Durham, NC, USA). The platform was used the Affymetrix HG-U133_Plus_2.0 Array. Expression values were calculated using Affymetrix GeneChip analysis software MAS 5.0. CEL files have been deposited to the Gene Expression Omnibus database (http://www.ncbi.nlm.nih.gov/geo) with the accession number GSE67342.

### Gene expression analysis with RT^2^ Profiler PCR Array

Following the analysis of transcriptomic data, commercial RT^2^ Profiler PCR Arrays were used for quantitation of 84 targeted genes involved in glucose metabolism pathways (Cat no. PAHS-006Z, QIAGEN, Germany) in LoVo cells treated with or without metformin for 8 and 24 h, which are independently different with those used for transcriptomic profiling. Briefly, the total RNA was extracted with RNeasy Mini Kit (Cat no. 74104, QIAGEN, Germany) and 1 μg of total RNA was subjected to first strand cDNA synthesis with RT^2^ HT First Strand Kit (Cat no. 330411, QIAGEN, Germany) according to the manufacturer’s instructions. The synthesized cDNA samples were analyzed with RT^2^ Profiler PCR Arrays in triplicate on ABI7500 system (ABI).

### Data analysis

For GC/TOFMS data analysis, the sample information, peak retention time, and peak area (quant mass) were included in the final data set. All those known artificial peaks, such as peaks caused by noise, column bleed, and the BSTFA derivatization procedure, were removed from the data set. The resulting data were normalized to the internal standard prior to statistical analysis. The normalized data were mean centered and unit variance scaled during chemometric data analysis in the SIMCA-p 13.0 Software package (Umetrics, Umeå, Sweden). The unsupervised multivariate statistic, principal component analysis (PCA) was first used to compare the metabolic profiles between groups. Differential variables were then selected with the criteria of variable importance in the projection (VIP > 1) in the partial least-squares-discriminant analysis (PLS-DA) model and p < 0.05 in a Student’s t-test. Compound identification was performed by comparing the mass fragments of interesting variables with NIST 05 standard mass spectral databases in NIST MS search 2.0 (NIST, Gaithersburg, MD) software at a similarity score of greater than 70%. The identified differential metabolites were then validated by using available reference compounds. The corresponding fold change shows how these selected differential metabolites varied between groups.

For LC/TOFMS data analysis, The resulting .d files were then centroided, deisotoped, and converted to mzData xml files using the MassHunter Qualitative Analysis Program (vB.03.01) (Agilent). Following the conversion, the xml files were analyzed using the open source XCMS package (v1.24.1) (http://metlin.scripps.edu), which runs in the statistical package R (v.2.12.1) (http://www.r-project.org), to pick, align, and quantify features (chromatographic events corresponding to specific m/z values and rention times). The software was used with default settings as described (http://metlin.scripps.edu) except for xset (bw = 5) and rector (plottype = “m”, family = “s”). The created .tsv file was opened using Excel software and saved as .xls file. The resulting data sheet was normalized to the internal standard and used for further analysis. Metabolite annotation was performed by comparing the accurate mass (m/z) and retention time (Rt) of reference standards in our in-house library and the accurate mass of compounds obtained from the web-based resources such as the Human Metabolome Database (http://www.hmdb.ca/) and The METLIN Metabolite Database (http://metlin.scripps.edu/).

For the microarray data analysis, only those probes with “perfect value” present in >2 samples (60%) in each group were applied in further analysis. Principal component analysis (PCA) was used to summarize gene expression profiles between groups. The SAM (Significance Analysis of Microarrays) method to evaluate statistical significance of changes in gene expression, and took the two-class unpaired analysis at false discovery rate (FDR) <0.01 and adopted a cutoff of 1.2-fold changes^15^. Gene Ontology (GO) enrichment analysis of the differentially expressed genes was performed using the Database for Annotation, Visualization, and Integrated Discovery (DAVID) (http://david.abcc.ncifcrf.gov/)^16^. To identify significant enrichment of GO terms, the expression analysis systematic explorer (EASE) score threshold in DAVID was set to ≥1.3 (p < 0.05). To analyze the relationship of differentially expressed genes with metabolic processes, the Paintomics (http://www.paintomics.org/cgi-bin/main2.cgi)^17^ was used to visualize the distribution of differentially expressed genes on known metabolic pathways in Kyoto Encyclopedia of Genes and Genomes (KEGG) (http://www.genome.jp/kegg/) database^18^. Hierarchical clustered heat maps were produced with Cluster 3.0 and TreeView software (M. B. Eisen Laboratory, Stanford University, Stanford, CA).

## Results

### Metformin dose- and time-dependently suppressed the proliferation of LoVo cells

**Figure 1:**
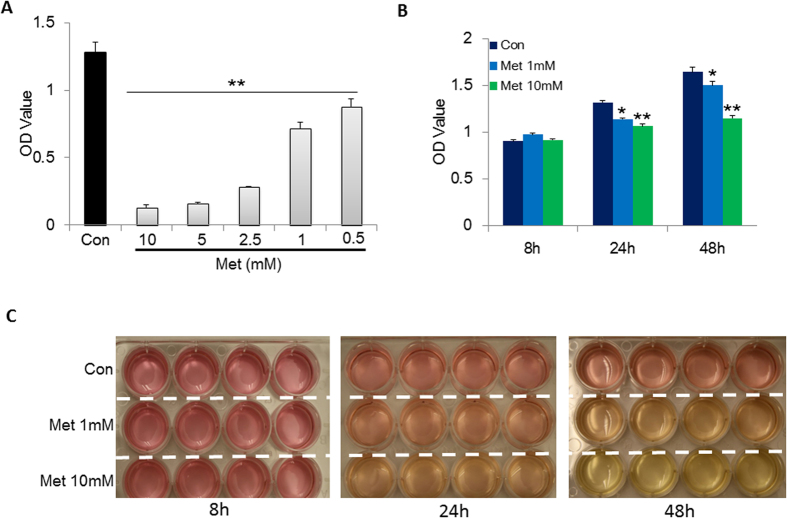
Metformin suppressed the proliferation of LoVo cells dose- and time-dependently. (**A**) Human-derived colon cancer cells, LoVo cells were cultured in 10% FBS DMEM and treated with or without metformin at a series of concentrations from 0.5 to 10 mM for 48 h. Then, cell viability was assayed with CCK-8 according to its instruction. (**B**) LoVo cells were treated with metformin for 8, 24 and 48 h at the concentrations of 1 and 10 mM, respectively. The cell number was evaluated with CCK-8. (**C**) The representative photograph of cell culture media treated with either 1 or 10 mM metformin for different time points. *P < 0.05, **P < 0.01 compared to corresponding control cells. Data are means ± S.E of at least two independent experiments with five replications for each group.

To determine an optimal concentration of metformin for suppressing proliferation of LoVo cells, we first treated LoVo cells with a series of concentrations of metformin from 0.5 to 10 mM for 48 h, and found that the proliferation of LoVo cells were significantly suppressed by metformin in a dose-dependent way (Fig. 1A). Then, we treated the LoVo cells with 1 mM and 10 mM metformin, respectively, and the cell viability was measured at 8, 24, and 48 h of treatment to characterize the time-dependent impacts of metformin. We observed that the proliferation of LoVo cells were significantly suppressed by metformin after 24 h treatment at both 1 and 10 mM concentrations, whereas there was no significant difference in cell viability among groups at 8 h (Fig. 1B). Meanwhile, the color of cell cultural media underwent obvious change in metformin treated wells at 24 and 48 h (Fig. 1B), which may due to the over-production of lactate induced by metformin.

### Metformin induced time-dependent metabolic reprogramming on LoVo cells

**Figure 2:**
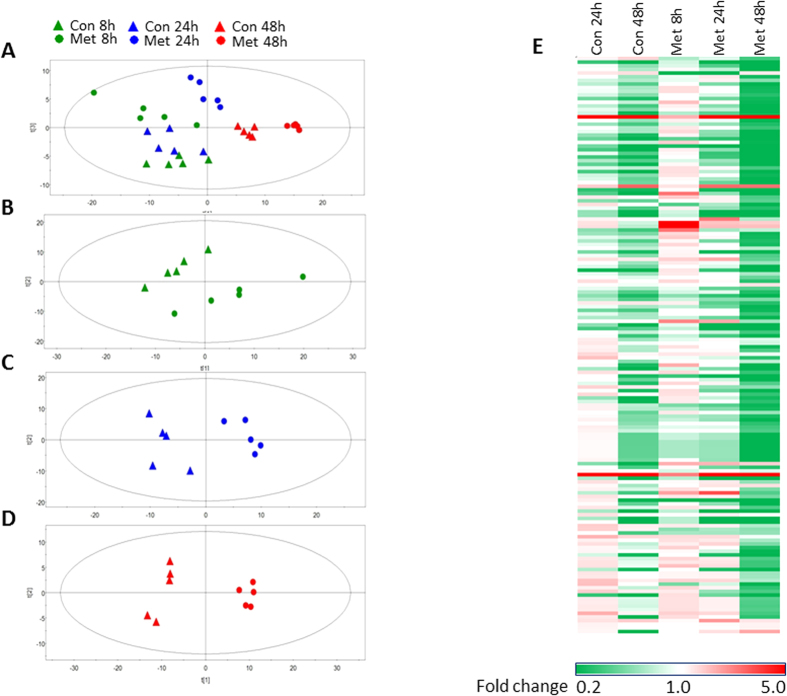
Metabolic profiling of LoVo cells treated with 10 mM metformin for different time points. (**A–D**) The PCA score plots for all groups, Con 8 h vs Met 8 h, Con 24 h vs Met 24 h, and Con 48 h vs Met 48 h, respectively. (**E**) The heatmap for the intensity variations of the total 158 metabolites identified with GC/TOFMS and LC/TOFMS together.

The metabolic profile was evaluated between control and metformin treated cells at three different time points using unsupervised statistics, PCA on the basis of 158 identified cellular metabolites from GC/TOFMS and LC/TOFMS. The PCA loading plots showed that the cell samples of con 8 h and con 24 h almost clustered together, but were clearly separated from those of con 48 h (Fig. 2A), suggesting the gradual alteration of cellular metabolites with culture time. A similar time-dependent metabolic alteration was also observed in metformin-treated cells (Fig. 2A), which was further characterized by individual comparisons between control and metformin groups at different time points (Fig. 2B–D). To further investigate the impacts of both culture time and metformin treatment, a heatmap among all groups was drawn on the basis of relative intensity of total 158 identified metabolites compared to con 8 h group. Generally, the amount of cellular metabolites was obviously downregulated with culture time either in control or metformin-treated cells. However, most metabolites in met 8 h group were upregulated in comparison with con 8 h, but were further depleted by metformin at 24 h and 48 h (Fig. 2E). These metabolic profiles indicate a time-dependent alteration in amounts of cellular metabolites resulted from culture time and/or metformin treatment.

To further investigate the metabolic impacts of metformin on LoVo cells, the supervised PLS-DA models were constructed between control and metformin-treated groups at different time points. The differential metabolites between groups were determined based on the criteria of VIP > 1 in the PLS-DA model and P < 0.05 in Student’s t-test. We identified 47, 45 and 66 differential metabolites between control and metformin-treated cells at 8, 24 and 48 h, respectively (Tables 1, 2, 3, [Table t1], [Table t2], [Table t2], [Table t2], [Table t2], [Table t3]), whereas the quality of PCA and PLS-DA models was summarized in Table 4. These differential metabolites are involved in the main energy metabolism pathways including amino acids, TCA cycle, carbohydrates, fatty acids, and nucleotides metabolism. In respect to the identified differential metabolites between control and metformin-treated cells at 8, 24 and 48 h, we observed that the exposure to metformin resulted in comprehensive impacts on cell metabolism, especially in energy metabolism, which is prior to the appearance of cell viability reduction. In metformin-treated cells, valine, leucine, isoleucine, and several other essential amino acids were transiently upregulated and finally depleted, suggesting the early suppression on protein synthesis by metformin. At 24 h, we observed that metformin-treated cells had almost five folds increase in amount of oxidized glutathione, and almost complete depletion of glutathione. The metformin-induced over-production of lactate is usually observed in diabetic patients. However, in this study we observed that the contents of cellular lactate were decreased by metformin at 24 and 48 h, which was accompanied with an increased lactate secretion into culture media (data not shown).

### Metformin altered the gene expression profile of LoVo cells time-dependently

**Figure 3:**
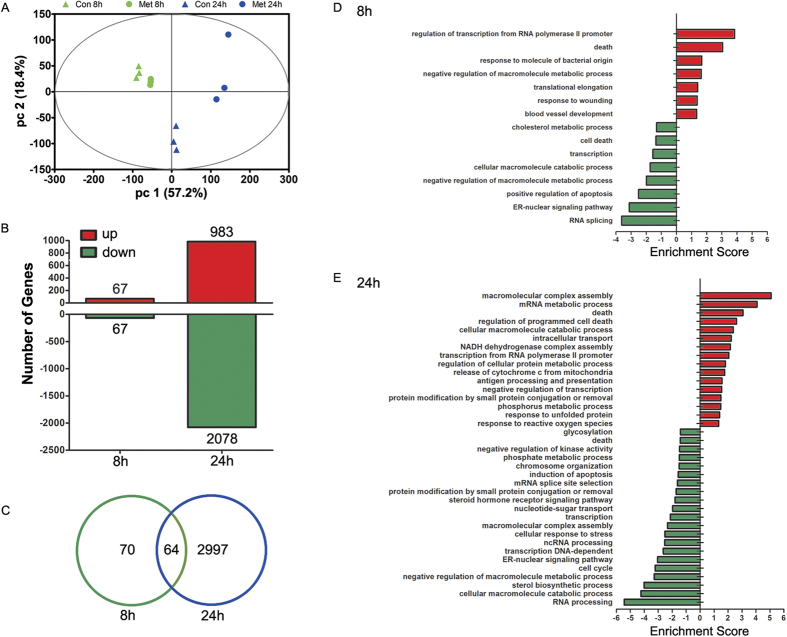
Transcriptomic profiling of LoVo cells treated with 10 mM metformin for 8 and 24 hours. (**A**) The PCA score plot of all analyzed transcripts of all 4 groups. (**B**) The summary of all up- and down-regulated genes by metformin treatment. (**C**) The number of altered genes by metformin treatment for either 8 or 24 h. (**D,E**) The GO enrichment analysis of altered genes by metformin treatment for either 8 or 24 h.

In addition to the metabolic profiling, the global gene expression of control and metformin-treated cells at 8 and 24 h was also analyzed for elucidating the transcriptional modulation of metformin. First, the transcriptomic profiling with all the detected probes was evaluated using PCA model. The PCA score plot showed that samples of control or metformin-treated cells at 8 h were relatively clustered together, whereas those at 24 h were distinctly separated, suggesting the time-related gene expression panel of control cells. Meanwhile, 24 h metformin treatment resulted in obvious separation with samples of con 24 h, which is consistent with their metabolic profiles (Fig. 3A). There were 134 (67 up-regulation and 67 down-regulation) and 3061 (983 up-regulation and 2078 down-regulation) differentially expressed genes between control and metformin-treated cells at 8 and 24 h, respectively (Fig. 3B). Most of the genes were uniquely regulated at the two time points, in which 64 genes were commonly modulated at both time points (Fig. 3C).

To classify these changes, we used GO enrichment analysis to test whether particular functional categories were enriched. At 8 h, four categories were enriched in the upregulated genes (Fig. 3D), including sterol biosynthetic process, RNA processing, response to calcium ion, and regulation of cellular protein metabolic process. Meanwhile, six categories were enriched in downregulated genes (Fig. 3D). Except glycerophospholipid metabolic process and positive regulation of transcription from RNA polymerase II promoter, other four categories were related with cell cycle process (Fig. 3D). At 24 h, upregulated genes were enriched in 16 categories (Fig. 3E), which related with cell activity (macromolecular complex assembly, intracellular transport, protein modification by small protein conjugation or removal, response to unfolded protein, and response to reactive oxygen species), cellular metabolic processes (cellular macromolecule catabolic process, NADH dehydrogenase complex assembly, regulation of cellular protein metabolic process, and phosphorus metabolic process), cell cycle (death, regulation of programmed cell death, and release of cytochrome c from mitochondria), regulation of transcription (mRNA metabolic process, transcription from RNA polymerase II promoter, and antigen processing and presentation), and immunity (antigen processing and presentation); downregulated genes were enriched in 21 categories (Fig. 3E), which related with cell activity (cellular response to stress, macromolecular complex assembly, steroid hormone receptor signaling pathway, protein modification by small protein conjugation or removal, negative regulation of kinase activity, and glycosylation), cellular metabolic processes (cellular macromolecule catabolic process, sterol biosynthetic process, negative regulation of macromolecule metabolic process, and phosphate metabolic process), cell cycle (cell cycle, induction of apoptosis, and death), and regulation of transcription (RNA processing, ER-nuclear signaling pathway, transcription, DNA-dependent, ncRNA processing, transcription, nucleotide-sugar transport, mRNA splice site selection, and chromosome organization).

### Metformin induced time-dependent transcriptional alteration in energy metabolism pathways on LoVo cells

**Figure 4:**
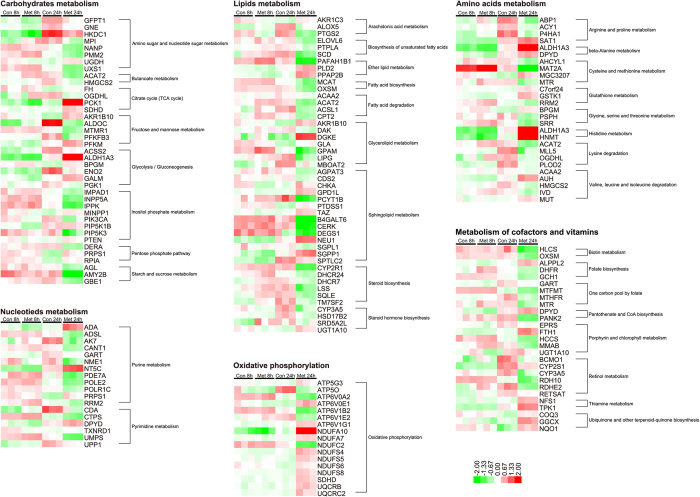
The heatmaps for altered expression of genes involved in energy metabolism pathways among all groups.

Metformin is a classical anti-diabetes drug by activating AMPK and its subsequent signaling pathways leading to the rebalancing of cell energy metabolism. Altered cell energy metabolism is a hallmark for tumor cells. To examine the comprehensive impacts on tumor cell energy metabolism of metformin, genes, which are mainly involved in cell energy metabolism pathways, were compared among groups including carbohydrates, lipids, amino acids, nucleotides, and vitamins metabolism (Fig. 4). Even though the metabolic profile of Met 8 h was significantly different from Con 8 h, the gene expression panels of energy metabolism pathways were similar between Con 8 h and Met 8 h (Fig. 4). A large number of genes were upregulated with culture time in Con 24 h group, however, most of the genes were downregulated by 24 h metformin treatment, except for several significantly upregulated genes such as phosphoenolpyruvate carboxykinase 1 (PCK1), aldehyde dehydrogenase 1 family, member A3 (ALDH1A3) in carbohydrates metabolism; phospholipase D2 (PLD2), lipid phosphate phosphohydrolase 3 (PPAP2B), diacylglycerol kinase (DGKE), sialidase 1 (NEU1) and sphingosine-1-phosphate phosphatase 1 (SGPP1) in lipids metabolism; spermidine/spermine N1-acetyltransferase 1 (SAT1), histamine N-methyltransferase (HNMT), and AU RNA binding protein/enoyl-CoA hydratase (AUH) in amino acids metabolism; adenosine deaminase (ADA), and 5′, 3′-nucleotidase, cytosolic (NT5C) in nucleotides metabolism; alkaline phosphatase, placental-like 2 (ALPPL2), ferritin, heavy polypeptide 1 (FTH1), NFS1 cysteine desulfurase (NFS1), and gamma-glutamyl carboxylase (GGCX) in vitamins metabolism (Fig. 4).

**Figure 5:**
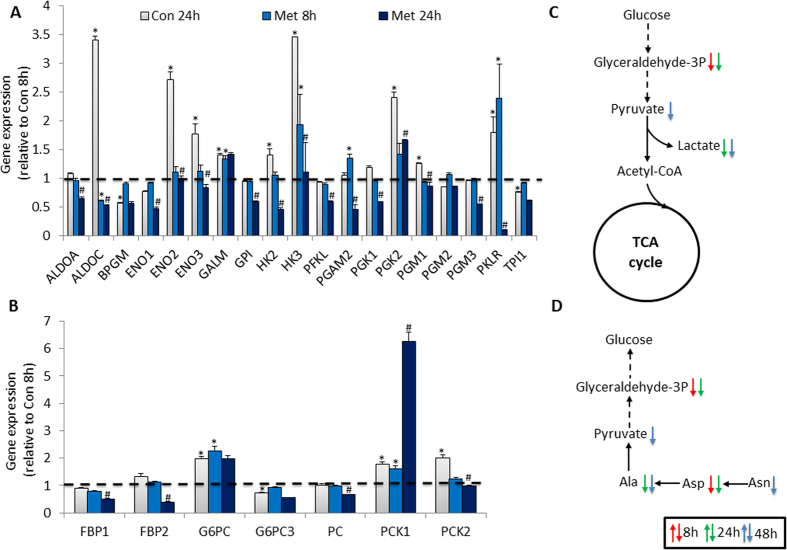
Summary of metabolites and gene expression alteration in glycolysis and gluconeogenesis processes. (**A,B**) The expression analysis of genes involved in glycolysis and gluconeogenesis processes by using RT^2^ Profiler Array. (**C,D**) Altered metabolites in glycolysis and gluconeogenesis processes by metformin treatment. Data are means ± S.E.M. of triplicates for each group. *indicates P < 0.05 compared to Con 8 h group, ^#^P < 0.05 compared to Con 24 h group with Student’s *t* test. The dashed and black arrows in metabolic processes represent indirect or direct reactions. The metabolites alteration at 8, 24 and 48 h are labeled with red, green or blue arrow, respectively.

**Figure 6:**
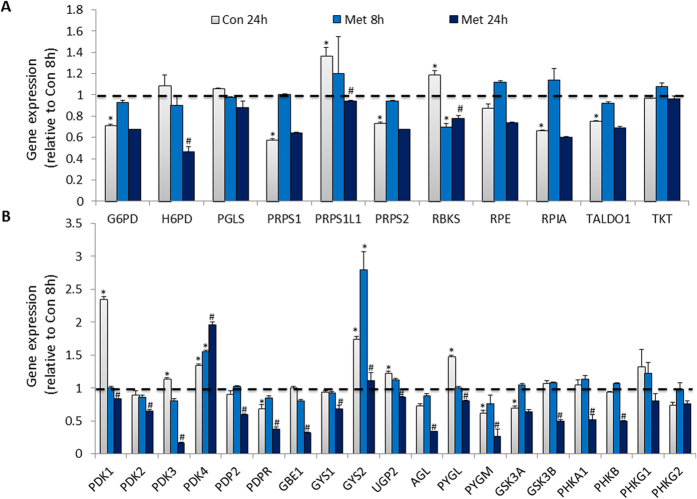
The expression analysis of genes involved in pentose phosphate pathway and regulation on glucose and glycogen metabolism. (**A**) The expression of genes in pentose phosphate pathway. (**B**) The expression of genes in regulation on glucose and glycogen metabolism. Data are means ± S.E.M. of triplicates for each group. *indicates P < 0.05 compared to Con 8 h group, ^#^P < 0.05 compared to Con 24 h group with Student’s *t* test.

To further characterize the time-dependent modulation on energy metabolism by metformin, we analyzed the expression of 84 genes in glucose metabolism pathways including genes in glycolysis, gluconeogenesis, regulation on glucose and gluconeogenesis, and TCA cycle processes. In general, the expression panel of the observed 81 genes (three genes were excluded due to very low expression level in all samples) in glucose metabolism pathways was consistent with the transcriptomic profiling. In glycolysis and gluconeogenesis pathway, a lot of genes were upregulated with culture time in control cells, but most of them were downregulated by metformin treatment, especially at 24 h (Fig. 5A,B). In gluconeogenesis pathway, although the expression of both phosphoenolpyruvate carboxykinase 1 (PCK1) and phosphoenolpyruvate carboxykinase 2 (PCK2) was upregulated with culture time in control cells, metformin treatment further stimulated the expression of PCK1, but suppressed PCK2 at 24 h (Fig. 5B). The transcriptional alteration was consistent with the observations of metabolic changes induced by metformin, in which several metabolites in glycolysis and gluconeogenesis processes were reduced in metformin-treated cells including pyruvate, lactate, glyceraldehyde-3P, alanine, and aspartic acid (Fig. 5C,D). Pentose phosphate pathway is an important metabolic pathway to generate NADPH and pentoses paralleling to glycolysis. In current study, we observed that most of the genes involved in pentose phosphate pathway was downregulated or unchanged at Con 24 h group compared to Con 8 h group, except for two upregulated genes at Con 24 h that is phosphoribosyl pyrophosphate synthetase 2 (PRPS2) and phosphoribosyl pyrophosphate synthetase 1-like 1 (PRPS1L1) which catalyze the synthesis of purines and pyrimidines, as well as ribonucleoside monophosphates. Although, 8 h metformin treatment did not induce obviously transcriptional changes among observed genes in pentose phosphate pathway, the expression panel of genes in Met 24 h group was similar to that of Con 24 h, except for the dramatic suppression of PRPS2, PRPS1L1, and hexose-6-phosphate dehydrogenase (H6PD) genes resulted from 24 h metformin treatment (Fig. 6A). In addition, genes which are regulators of glycolysis and gluconeogenesis pathways were also analyzed. We observed that most genes were not changed at Met 8 h group, but were significantly suppressed at Met 24 h group compared to Con 8 h or Con 24 h group respectively (Fig. 6B).

**Figure 7:**
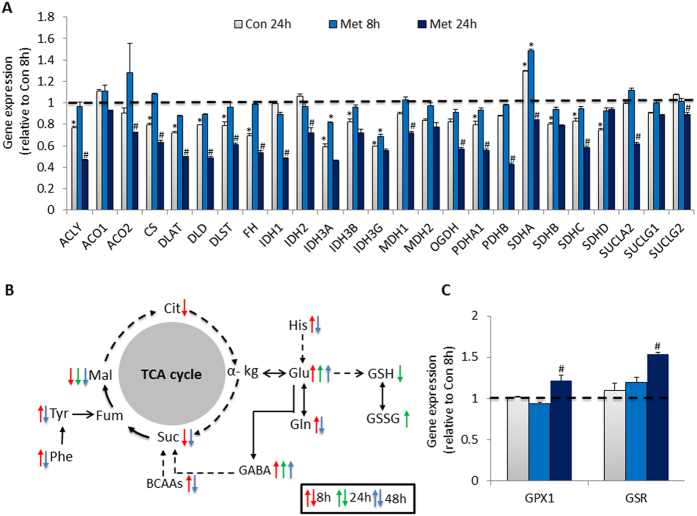
Summary of metabolites and gene expression alteration in TCA cycle. (**A**) The expression analysis of genes involved in TCA cycle by using RT^2^ Profiler Array. (**B**) Altered metabolites in TCA cycle by metformin treatment. (**C**) The expression of genes in modulating the conversion between reduced and oxidized glutathione in microarray. Data are means ± S.E.M. of triplicates for each group. *indicates P < 0.05 compared to Con 8 h group, ^#^P < 0.05 compared to Con 24 h group with Student’s *t* test. The dashed and black arrows in metabolic processes represent indirect or direct reactions. The metabolites alteration at 8, 24 and 48 h are labeled with red, green or blue arrow, respectively.

TCA cycle is the key process for tumor cells to generate ATP and intermediates for macromolecule biosynthesis (PNAS, 27). In this study, we found that about half of observed genes in TCA cycle was downregulated at con 24 h, and most of them was further reduced by metformin treatment (Fig. 7A). At metabolic level, several intermediates of TCA cycle were reduced in metformin-treated cells including citrate, malate, fumarate, and succinate. Meanwhile, the levels of some amino acids were significantly altered in metformin-treated cells such as increasing of tyrosine, phenylalanine, BCAAs, GABA, glutamate, glutamine and histidine at 8 h, but decreasing at 24 or 48 h (Fig. 7B). These amino acids play important roles in energy metabolism by conversion to intermediates of TCA cycle directly or indirectly. In addition to energy metabolism, the intracellular redox status is also important for tumor cell proliferation and survivor. In current study, we observed that 24 h treatment of metformin resulted in significant depletion of reduced glutathione and increasing of oxidized glutathione, suggesting the imbalance of intracellular redox status induced by metformin. Consistently, we found the expression of Glutathione peroxidase (GPX) 1 and glutathione reductase (GSR) genes were significantly upregulated by 24 h metformin treatment (Fig. 7C), which mediate the conversion between reduced- and oxidized- glutathione.

## Discussion

Our current study demonstrated that metformin treatment resulted in a comprehensive metabolic and transcriptional alteration on LoVo cells, which are prior to the phenotypic changes in cell viability. There are about 50 differential metabolites between metformin and control cells at 8, and 24 hours, whereas 66 at 48 hours. Meanwhile, more than 130 and 3000 differently expressed genes are observed in metformin treated cells at 8 and 24 hours comparing to corresponding control cells, respectively. In addition, the subsequent analysis of genes in glucose metabolism pathways confirms the time-dependent transcriptional modulation of metformin on LoVo cells.

LoVo cells are derived from a colorectal cancer patient who is classified as Duke’s type C and grade IV. Since different types of cancer cells usually present different genetic and metabolic phenotypes leading to variable responses towards identical treatment, in current study, the inhibitory effect of metformin was also validated in another two colorectal cancer cells, Caco-2 and HT-29, in addition to LoVo cells. The results showed consistent reduction in cell viability by metformin treatment at different concentrations (Fig S1). However, a more satisfactory time- and dose-dependent suppressive effect was only observed in metformin-treated LoVo cells in our study. Meanwhile, the antitumor effect of metformin has been investigated in several other colorectal cancer cells such as HCT116, HT-29, and SW620^19^,^20^,^21^, whereas the suppressive effect of metformin on LoVo cells was little known. Accordingly, the LoVo cells were chosen for the transcriptomic and metabolic profiling in our current study.

The dysregulation of energy metabolism is the common pathophysiological basis for both metabolic diseases and tumors^22^. In recent years, a huge body of evidence has highlighted the therapeutic potential of tumors by modulating tumor cell energy metabolism^23^. Metformin is a potent activator of AMPK, the most important intracellular energy sensor. Meanwhile, metformin is also proposed to function as a mitochondrial poisoner by suppressing the activity of mitochondrial complex I, leading to the imbalance of AMP : ATP ratio, which is monitored by AMPK or activates AMPK vice versa^8^. Accordingly, the preventive or therapeutic effect on tumors of metformin is associated with its modulation on tumor cell energy metabolism, in addition to its regulation on tumor-related signaling pathways. In our current study, although no significant difference was observed in cell viability at 8 h of metformin treatment, 47 differential metabolites were identified between Met 8 h and Con 8 h group, and most of them were up-regulated in metformin-treated cells. Valine, leucine and isoleucine are BCAAs, which are the building-block for protein synthesis. In our study, the concentrations of these BCAAs were higher at 8 h, but depleted at 48 h in metformin-treated cells, suggesting the suppression of tumor cell protein synthesis by metformin is prior to the reduction of cell viability, which is consistent with the proposed anti-tumor function of metformin by inhibiting tumor cell protein synthesis^24^.

Tumor cells are usually characterized with aerobic glycolysis (Warburg effect) in glucose metabolism leading to overproduction of lactate from pyruvate even though in the presence of sufficient oxygen^25^. Although the aerobic glycolysis is inefficient in ATP production, it provides advantages for tumor cell proliferation by producing large amount of required biomass for tumor cell division such as nucleotides, amino acids, and lipids^25^,^26^. Metformin is observed to reduce the intermediates of TCA cycle, and pyruvate in cancer stem cells^12^, but not in prostate cancer cells, which are more dependent on energy fueling of glutamine metabolism^27^. Although metformin is effective in suppression of various tumor cells, these observations indicate that the metabolic regulation of metformin on tumor cells may be cell type-specific. In our study, metformin treatment reduced the contents of citrate, succinate and malate in TCA cycle prior to the cell viability reduction. TCA cycle is the key process for energy metabolism in mitochondria, which orchestrates the energy metabolism among glucose, fatty acids and amino acids metabolism. The reduced intermediates in TCA cycle suggested the inhibition of energy metabolism on LoVo cells by metformin. Meanwhile, parts of metabolites in glycolysis and gluconeogenesis such as pyruvate, lactate, glyceraldehyde-3P, alanine, and aspartic acid were also downregulated by metformin, which suggested the suppression of glucose metabolism on LoVo cells. At the transcriptional level, most of the genes involved in glycolysis were upregulated with culture time in control cells, but were largely downregulated by metformin treatment, especially at 24 h. For example, phosphofructokinase (PFK), pyruvate kinase (PK), and hexokinase (HK) are the genes for encoding the rate-limiting enzymes in glycolysis, which were universally suppressed by 24 h metformin treatment implying the inhibition of glycolysis by metformin. The overproduction of lactate is usually expected in metformin treatment^28^. In tumor cells, pyruvate is mainly metabolized into lactate in aerobic glycolysis, whereas metformin treatment could increase the secretion of lactate into culture medium in tumor cells^29^. In our current study, we observed that the concentration of cellular lactate was time-dependently depleted in metformin-treated cells, which was accompanied with a mild increase of lactate in culture medium (data not shown) suggesting the increased secretion of lactate into culture medium in metformin-treated cells.

The genes responsible for regulation of gluconeogenesis were differently modulated by metformin treatment. Phosphoenolpyruvate carboxykinases (PCKs), fructose-1,6-bisphosphatase 1,2 (FBP1, FBP2), and pyruvate carboxylase (PC) are the main control for gluconeogenesis. We observed that the expression of FBP1, FBP2, PC and PCK2 (mitochondrial) genes was significantly suppressed by metformin at 24 h, except for the obvious up-regulation of PCK1 (soluble). PCK1 (soluble) and PCK2 (mitochondrial) encode a cytosolic or mitochondrial enzyme catalyzing the formation of phosphoenolpyruvate from oxaloacetate along with GTP. In current study, the paradoxical modulation on these two genes by metformin suggested that metformin could differently regulate the expression of cytosolic or mitochondrial isoforms of particular genes. Since metformin can inhibit protein synthesis and gluconeogenesis in cells by activating AMPK^30^, the transcriptional and metabolic results consistently suggested the time-dependent suppression of gluconeogenesis by metformin.

In tumor cells, TCA cycle is also an important source of biosynthetic precursors, in addition to the production of ATP^26^. For example, citrate in mitochondria can be transported into cytoplasm for lipogenesis, which is critical for tumor cell proliferation. ATP citrate lyase (ACLY) is the primary enzyme responsible for the synthesis of cytosolic acetyl-CoA and oxaloacetate from citrate and CoA, which is recognized as a potential antitumor therapeutic target^31^. In our current study, we observed that ACLY was significantly suppressed by 24 h metformin treatment, as well as the decreased level of cellular citrate in metformin-treated cells at 8 h. Moreover, another two intermediates of TCA cycle were also reduced by 8 h metformin treatment, whereas the transcriptional suppression of most genes in regulation of TCA cycle was only present after 24 h metformin treatment. Pyruvate dehydrogenase (PDH) complex is a critical mitochondrial enzyme complex that catalyzes the conversion of pyruvate to acetyl-CoA and CO_2_, and provides the link between glycolysis and TCA cycle. The activity of PDH is inhibited by pyruvate dehydrogenase kinase 4 (PDK4) leading to the suppression of pyruvate conversion to acetyl-CoA^32^. Metformin has been observed to suppress the activity of PDH resulting in the suppression on glycolysis in cancer cells^33^. In our current study, we found that the two isoforms of PDH, pyruvate dehydrogenase (lipoamide) alpha 1 (PDHA1) and pyruvate dehydrogenase (lipoamide) beta (PDHB) were down-regulated by 24 h metformin treatment, while the PDK4 was time-dependently up-regulated by metformin at both 8 and 24 h suggesting that metformin may suppress the expression of PDH by up-regulating PDK4 expression. However, the expression of PDK1, PDK2 and PDK3 was down-regulated by 24 h metformin treatment. It is reported that the overexpression of PDK3 is positively associated with severity of cancer and negatively associated with disease-free survival in colon cancer patients. Moreover, the expression of PDK3 is controlled by HIF-1αand contributes to hypoxia-induced drug resistance in colon cancer cells^34^. Accordingly, the suppression on PDK3 by metformin may contribute to its anti-tumor activity in LoVo cells.

In addition to the glucose-derived energy supply, glutamine/glutamate also fuels tumor cell through conversion toα-ketoglutarate in TCA cycle. In previous report, metformin has been observed to reduce levels of both glutamine and glutamate in breast cancer stem cells, and no defect in glutamine transport across the cell membrane^12^. However, we observed that glutamate was increased by metformin treatment at all three time points, while glutamine was increased at 8 h and reduced at 48 h. We hypothesized that metformin blocked the conversion of glutamate to α-ketoglutarate, which could reduce the glutamine/glutamate-derived energy supply via TCA cycle. In addition to the role in energy supply, glutamate is one of the components of glutathione, which modulates the balance of intracellular redox status via conversion between reduced- and oxidized- glutathione. The imbalance of intracellular redox status leads to oxidative stress, which plays critical roles in tumor cells proliferation and apoptosis. In oesophageal squamous cell carcinoma cell lines, metformin is observed to reduce the cell proliferation, and protect the cells against cisplatin toxicity because of the induction of glycolysis and reduced intracellular thiols, while the glutathione synthesis inhibitor can ablate the protective effect of metformin^35^. However, in our current study, we found that 24 h metformin treatment dramatically depleted the reduced glutathione and increased oxidized glutathione, suggesting the imbalance between intracellular redox status induced by metformin on LoVo cells.

In summary, our current study indicates that metformin treatment produces comprehensive metabolic and transcriptional alteration on LoVo cells, which is time-dependent and prior to the occurrence of cell viability reduction. Moreover, the tumor cell energy metabolism pathway is one of the main targets for metformin activity.

## Additional Information

**How to cite this article**: He, J. *et al.* Metformin suppressed the proliferation of LoVo cells and induced a time-dependent metabolic and transcriptional alteration. *Sci. Rep.* **5**, 17423; doi: 10.1038/srep17423 (2015).

## Supplementary Material

Supplementary Information

## Figures and Tables

**Table 1 t1:** The observed differential metabolites between control and metformin-treated cells at 8 h.

Metabolite	RT_Min	Mean ± SD		P value^a^	VIP	Fold change (Met8 h/Con8 h)
		Con 8 h	Met 8 h			
Glyceraldehyde-3P^GC^	6.61	0.0437 ± 0.0136	0.0041 ± 0.0014	0.000	1.42	0.09
Valine^GC^	7.71	0.1159 ± 0.0157	0.1883 ± 0.0426	0.007	1.20	1.63
Leucine^GC^	8.50	0.1122 ± 0.0156	0.1820 ± 0.0386	0.006	1.22	1.62
Isoleucine^GC^	8.81	0.0781 ± 0.0337	0.1605 ± 0.0399	0.008	1.19	2.05
Succinate^GC^	9.01	0.0073 ± 0.0009	0.0055 ± 0.0012	0.026	1.20	0.75
Serine^GC^	9.74	0.1480 ± 0.01880	0.2167 ± 0.0430	0.011	1.16	1.46
Threonine^GC^	10.11	0.0952 ± 0.0128	0.1595 ± 0.0285	0.002	1.29	1.68
Malate^GC^	11.38	0.0184 ± 0.0030	0.0090 ± 0.0020	0.000	1.43	0.49
Methionine^GC^	11.75	0.0129 ± 0.0017	0.0239 ± 0.0046	0.001	1.32	1.85
Aspartic acid^GC^	11.78	0.9564 ± 0.1325	0.2710 ± 0.0570	0.000	1.50	0.28
5-oxoproline^GC^	11.79	0.8862 ± 0.1427	1.2261 ± 0.2588	0.033	1.05	1.38
Citrulline^GC^	12.89	0.0117 ± 0.0019	0.0186 ± 0.0036	0.005	1.22	1.58
Glutamate^GC^	12.95	0.2884 ± 0.0515	0.4623 ± 0.1111	0.013	1.15	1.60
Phenylalanine^GC^	13.05	0.0564 ± 0.0084	0.0863 ± 0.0183	0.011	1.17	1.53
D-Xylose^GC^	13.76	0.0102 ± 0.0017	0.0154 ± 0.0046	0.044	1.00	1.51
Glutamine^GC^	14.67	0.2012 ± 0.0457	0.6447 ± 0.1730	0.001	1.35	3.20
Hypoxanthine^GC^	15.01	0.0233 ± 0.0037	0.0445 ± 0.0085	0.001	1.33	1.91
Citrate^GC^	15.23	0.0066 ± 0.0015	0.0030 ± 0.0022	0.016	1.21	0.46
Lysine^GC^	16.22	0.0256 ± 0.0025	0.0437 ± 0.0114	0.009	1.19	1.71
Histidine^GC^	16.23	0.0101 ± 0.0012	0.0195 ± 0.0056	0.007	1.21	1.92
Tyrosine^GC^	16.40	0.0921 ± 0.0139	0.1549 ± 0.0322	0.004	1.25	1.68
Xanthine^GC^	17.28	0.0015 ± 0.0002	0.0028 ± 0.0008	0.009	1.18	1.82
Palmitic acid^GC^	17.34	0.0510 ± 0.0052	0.0723 ± 0.0155	0.020	1.11	1.42
Kynurenine^GC^	19.23	0.0046 ± 0.0006	0.0070 ± 0.0015	0.014	1.14	1.50
9-octadecenoate^GC^	19.29	0.0174 ± 0.0018	0.0246 ± 0.0047	0.013	1.15	1.41
Tryptophan^GC^	19.57	0.0076 ± 0.0013	0.0128 ± 0.0030	0.007	1.20	1.68
Spermine^GC^	23.63	0.0009 ± 0.0003	0.0027 ± 0.0007	0.001	1.32	2.96
Allothreonine^LC^	3.44	2.1551 ± 0.3272	3.6596 ± 0.7127	0.003	1.27	1.70
GABA^LC^	3.48	4.6049 ± 0.7340	7.7433 ± 1.6965	0.005	1.23	1.68
Galactonic acid^LC^	3.48	2.2804 ± 0.4442	3.6633 ± 0.8118	0.010	1.17	1.61
Delta-hydroxylysine^LC^	3.52	0.1270 ± 0.0190	0.0734 ± 0.0213	0.003	1.35	0.58
hydroxyacetic acid^LC^	3.55	0.4555 ± 0.0818	0.6542 ± 0.1329	0.022	1.10	1.44
Ornithine^LC^	3.60	1.9194 ± 0.2950	1.3819 ± 0.2607	0.016	1.23	0.72
5-Aminolevulinic acid^LC^	3.60	34.7644 ± 4.9563	25.0486 ± 4.7538	0.013	1.24	0.72
Aminoadipic acid^LC^	3.64	0.4158 ± 0.0509	0.9531 ± 0.1776	0.000	1.39	2.29
Glyceric acid^LC^	3.73	0.1207 ± 0.0120	0.3686 ± 0.0701	0.000	1.42	3.05
2,3,4-Trihydroxybutyric acid^LC^	3.81	5.4045 ± 0.8624	8.7672 ± 1.5911	0.003	1.26	1.62
Acetylcarnitine^LC^	3.82	0.8168 ± 0.1714	1.2347 ± 0.1398	0.003	1.27	1.51
5-Aminopentanoic acid^LC^	3.84	6.7259 ± 1.0422	10.4121 ± 1.5679	0.002	1.28	1.55
Deoxyguanosine^LC^	3.85	0.2522 ± 0.0383	0.1574 ± 0.0284	0.002	1.35	0.62
Betaine^LC^	3.87	0.4511 ± 0.0792	0.7131 ± 0.1368	0.006	1.21	1.58
D-Ribose^LC^	4.08	0.0577 ± 0.0084	0.1046 ± 0.0214	0.002	1.29	1.81
Uracil^LC^	5.08	0.1132 ± 0.0232	0.1677 ± 0.0472	0.049	1.00	1.48
L-alloisoleucine^LC^	8.51	15.5642 ± 2.3698	26.255 ± 4.2595	0.001	1.32	1.69
Inosine^LC^	9.78	8.6605 ± 1.4267	12.4076 ± 2.3258	0.015	1.13	1.43
Methylmaleic acid^LC^	11.43	0.1491 ± 0.0222	0.2352 ± 0.0440	0.005	1.24	1.58
N-acetylserotonin^LC^	16.47	0.0438 ± 0.0162	0.0710 ± 0.0116	0.016	1.12	1.62

^GC^represents metabolites identified with GC/TOFMS.

^LC^represents metabolites identified with LC/TOFMS.

^a^the P value was obtained with Student’s *t* test.

**Table 2 t2:** The observed differential metabolites between control and metformin-treated cells at 24 h.

Metabolite	RT_Min	Mean ± SD		P value^a^	VIP	Fold change (Met24 h/Con24 h)
		Con 24 h	Met 24 h			
Lactate^GC^	5.45	1.3143 ± 0.5747	0.3240 ± 0.0499	0.005	1.25	0.25
Alanine^GC^	6.05	0.6414 ± 0.2393	0.3048 ± 0.0393	0.015	1.15	0.48
Glyceraldehyde-3P^GC^	6.61	0.0640 ± 0.0148	0.0020 ± 0.0009	0.000	1.49	0.03
Aspartic acid^GC^	11.78	0.5361 ± 0.2581	0.1160 ± 0.0431	0.000	1.22	0.22
4-hydroxy-proline^GC^	11.86	0.0059 ± 0.0023	0.0024 ± 0.0005	0.011	1.18	0.41
Glutamate^GC^	12.95	0.1223 ± 0.0556	0.2268 ± 0.0411	0.010	1.20	1.85
Ornithine^GC^	15.17	0.0077 ± 0.0057	0.0161 ± 0.0054	0.043	1.02	2.09
Myristic acid^GC^	15.32	0.0029 ± 0.0005	0.0020 ± 0.0006	0.033	1.06	0.69
D-Erythrotetrofuranose^GC^	15.34	0.3538 ± 0.1454	0.1015 ± 0.0207	0.005	1.25	0.29
1,5-anhydroglucitol^GC^	15.43	0.0226 ± 0.0095	0.0058 ± 0.0008	0.004	1.26	0.26
Adenine^GC^	15.65	0.0076±0.0036	0.0187 ± 0.0033	0.001	1.36	2.45
Pantothenic acid^GC^	16.55	0.0010 ± 0.0003	0.0004 ± 0.0001	0.004	1.27	0.42
Kynurenine^GC^	19.23	0.0010±0.0003	0.0017 ± 0.0002	0.003	1.31	1.74
Spermine^GC^	23.63	0.0010 ± 0.0005	0.0022 ± 0.0005	0.005	1.25	2.15
inosine 5′-monophosphate^GC^	24.28	0.0154 ± 0.0074	0.0041 ± 0.0011	0.010	1.19	0.26
Adenosine 5-monophosphate^GC^	26.56	0.0056 ± 0.0018	0.0019 ± 0.0016	0.009	1.20	0.34
Cholesterol^GC^	27.52	0.3037 ± 0.0318	0.2310 ± 0.0294	0.006	1.25	0.76
Hypotaurine^LC^	3.05	2.0930 ± 0.2988	0.9156 ± 0.1051	0.000	1.47	0.44
Taurine^LC^	3.07	0.5057 ± 0.0459	0.4231 ± 0.0452	0.021	1.13	0.84
D-fructuose^LC^	3.37	0.5230 ± 0.0472	0.4463±0.0465	0.032	1.07	0.85
Glycine^LC^	3.39	0.2408 ± 0.0656	0.1015 ± 0.0086	0.002	1.33	0.42
Allothreonine^LC^	3.44	3.4034 ± 0.2244	2.1893 ± 0.2177	0.000	1.48	0.64
GABA^LC^	3.48	4.137 ± 0.9823	5.8457 ± 0.4433	0.008	1.22	1.41
D-Glucuronic acid^LC^	3.48	0.7626 ± 0.3637	0.2753 ± 0.0951	0.020	1.11	0.36
Galactonic acid^LC^	3.48	2.2267 ± 0.2468	1.2117 ± 0.0448	0.000	1.48	0.54
Threonine^LC^	3.49	0.9969 ± 0.1429	0.5629 ± 0.0957	0.001	1.39	0.56
Delta-hydroxylysine^LC^	3.52	0.1686 ± 0.0166	0.0600 ± 0.0069	0.000	1.52	0.36
Carnitine^LC^	3.52	2.0737 ± 0.1821	0.9587 ± 0.0803	0.000	1.51	0.46
Creatinine^LC^	3.59	0.3667 ± 0.0406	0.2182 ± 0.0240	0.000	1.44	0.59
5-Aminolevulinic acid^LC^	3.60	41.336 ± 3.4185	23.0472 ± 1.8495	0.000	1.50	0.56
Proline^LC^	3.63	17.9432 ± 1.5773	9.1569 ± 0.8769	0.000	1.50	0.51
Aminoadipic acid^LC^	3.64	0.2530 ± 0.0301	0.9234 ± 0.0951	0.000	1.53	3.65
D-Xylose^LC^	3.67	0.3866 ± 0.0388	0.2091 ± 0.0150	0.000	1.49	0.54
N-Acetylneuraminic acid^LC^	3.75	1.0740 ± 0.2292	0.2525 ± 0.0250	0.000	1.46	0.24
2,3,4-Trihydroxybutyric acid^LC^	3.81	7.1387 ± 1.0684	5.7593 ± 0.7837	0.048	1.00	0.81
Oxidized glutathione^LC^	3.82	0.9742 ± 0.5264	4.7470 ± 0.4473	0.000	1.52	4.87
Glutathione^LC^	3.83	19.7071 ± 14.5982	0.4223 ± 0.0468	0.018	1.12	0.02
Gluconolactone^LC^	3.84	0.7119 ± 0.1774	0.0639 ± 0.0216	0.000	1.47	0.09
Deoxyguanosine^LC^	3.85	0.4581 ± 0.0517	0.1808 ± 0.0321	0.000	1.50	0.39
Malate^LC^	3.86	7.3946 ± 0.4779	5.0875 ± 1.8503	0.027	1.08	0.69
Cytosine^LC^	4.17	1.2571 ± 0.2712	0.8159 ± 0.1184	0.010	1.18	0.65
3-Hydroxypropionic Acid^LC^	5.11	127.9337 ± 24.6667	49.0766 ± 2.8241	0.000	1.44	0.38
Inosine^LC^	9.78	16.1774 ± 3.8742	5.6126 ± 1.9391	0.001	1.38	0.35
Tryptophan^LC^	14.03	1.7719 ± 0.1565	1.2871 ± 0.1394	0.001	1.37	0.73
N-acetylserotonin^LC^	16.47	0.0647 ± 0.0083	0.0477 ± 0.0060	0.006	1.24	0.74

^GC^represents metabolites identified with GC/TOFMS.

^LC^represents metabolites identified with LC/TOFMS.

^a^the P value was obtained with Student’s *t* test.

**Table 3 t3:** The observed differential metabolites between control and metformin-treated cells at 48 h.

Metabolite	RT_Min	Mean ± SD		P value^a^	VIP	Fold change (Met48 h/Con48 h)
		Con 48 h	Met 48 h			
Pyruvate^GC^	5.27	0.0059 ± 0.0015	0.0014 ± 0.0010	0.004	1.00	0.23
Lactate^GC^	5.45	0.3588 ± 0.0275	0.0354 ± 0.0203	0.000	1.20	0.10
Alanine^GC^	6.05	0.4318 ± 0.0506	0.0516 ± 0.0101	0.000	1.19	0.12
Beta-alanine^GC^	7.30	0.0159 ± 0.0041	0.0030 ± 0.0008	0.001	1.06	0.19
Valine^GC^	7.71	0.0835 ± 0.0099	0.0278 ± 0.0052	0.000	1.16	0.33
Leucine^GC^	8.50	0.0849 ± 0.0137	0.0284 ± 0.0057	0.000	1.12	0.33
Isoleucine^GC^	8.81	0.0611 ± 0.0067	0.0175 ± 0.0023	0.000	1.17	0.29
Proline^GC^	8.85	0.2134 ± 0.0228	0.0488 ± 0.0072	0.000	1.18	0.23
Succinate^GC^	9.01	0.0014 ± 0.0002	0.0004 ± 0.0002	0.001	1.04	0.27
Threonine^GC^	10.11	0.0668 ± 0.0108	0.0104 ± 0.0023	0.000	1.16	0.16
4-hydroxy-proline^GC^	11.86	0.0029 ± 0.0003	0.0002 ± 0.0001	0.000	1.20	0.09
Creatine^GC^	12.24	0.1928 ± 0.0281	0.0471 ± 0.0075	0.000	1.15	0.24
Phenylalanine^GC^	13.05	0.0342 ± 0.0071	0.0135 ± 0.0027	0.002	1.04	0.40
Asparagine^GC^	13.55	0.0463 ± 0.0093	0.0143 ± 0.0038	0.001	1.09	0.31
Hypoxanthine^GC^	15.01	0.0265 ± 0.0046	0.0102 ± 0.0022	0.000	1.09	0.39
D-Erythrotetrofuranose^GC^	15.34	0.1213 ± 0.0182	0.0022 ± 0.0006	0.000	1.18	0.02
1,5-anhydroglucitol^GC^	15.43	0.0073 ± 0.0010	0.0001 ± 0.0000	0.000	1.19	0.01
Lysine^GC^	16.22	0.0148 ± 0.0013	0.0060 ± 0.0022	0.000	1.11	0.41
Histidine^GC^	16.23	0.0081 ± 0.0010	0.0026 ± 0.0005	0.000	1.15	0.32
Tyrosine^GC^	16.40	0.0614 ± 0.0138	0.0243 ± 0.0030	0.002	1.02	0.40
Pantothenic acid^GC^	16.55	0.0005 ± 0.0001	0.0000 ± 0.0000	0.000	1.19	0.10
Myo-Inositol^GC^	18.24	5.8843 ± 0.6433	1.6396 ± 0.3448	0.000	1.17	0.28
Kynurenine^GC^	19.23	0.0007 ± 0.0001	0.0002 ± 0.0001	0.000	1.10	0.36
Tryptophan^GC^	19.57	0.0085 ± 0.0012	0.0025 ± 0.0006	0.000	1.15	0.30
2-Monostearin^GC^	22.25	0.0008 ± 0.0002	0.0004 ± 0.0002	0.001	1.05	0.53
Inosine^GC^	22.49	0.0474 ± 0.0126	0.0104 ± 0.0037	0.003	1.02	0.22
Adenosine^GC^	22.86	0.0012 ± 0.0003	0.0004 ± 0.0002	0.001	1.04	0.30
Inosine 5′-monophosphate^GC^	24.28	0.0040 ± 0.0012	0.0005 ± 0.0001	0.001	1.07	0.12
Adenosine 5-monophosphate^GC^	26.56	0.0063 ± 0.0017	0.0005 ± 0.0002	0.002	1.02	0.08
D-fructose^LC^	3.37	0.2804 ± 0.0294	0.1680 ± 0.0083	0.000	1.17	0.60
Glycine^LC^	3.39	0.1144 ± 0.0128	0.0390 ± 0.0074	0.000	1.18	0.34
Glutamine^LC^	3.43	0.2629 ± 0.0224	0.1598 ± 0.0350	0.000	1.11	0.61
Allothreonine^LC^	3.44	2.9290 ± 0.1941	0.7707 ± 0.0699	0.000	1.20	0.26
Taurine^LC^	3.45	4.8359 ± 0.4289	1.9141 ± 0.1473	0.000	1.19	0.40
GABA^LC^	3.48	1.3601 ± 0.2207	2.0488 ± 0.1230	0.002	1.04	1.51
Galactonic acid^LC^	3.48	0.6262±0.0611	0.4129 ± 0.0484	0.000	1.11	0.66
Glutamate^LC^	3.51	0.8393 ± 0.1180	1.2344 ± 0.0529	0.001	1.04	1.47
Delta-hydroxylysine^LC^	3.52	0.0990 ± 0.0095	0.0289 ± 0.0047	0.000	1.19	0.29
Carnitine^LC^	3.52	1.1705 ± 0.1292	0.4121 ± 0.0315	0.000	1.19	0.35
N-Acetylneuraminic acid^LC^	3.58	8.6904 ± 0.7124	4.6462 ± 0.5748	0.000	1.18	0.53
Creatinine^LC^	3.59	0.1460 ± 0.0208	0.0615 ± 0.0310	0.000	1.13	0.42
Ornithine^LC^	3.60	0.9403 ± 0.0848	0.3607 ± 0.0229	0.000	1.20	0.38
5-Aminolevulinic acid^LC^	3.60	17.1012 ± 1.6318	6.5918 ± 0.4471	0.000	1.19	0.39
Aminoadipic acid^LC^	3.64	0.0683 ± 0.0248	0.7573 ± 0.0516	0.000	1.20	11.09
D-Xylose^LC^	3.67	0.1676 ± 0.0077	0.1282 ± 0.0100	0.000	1.15	0.76
Glyceric acid^LC^	3.73	3.7230 ± 1.4490	6.9782 ± 0.9533	0.001	1.08	1.87
Methylcysteine^LC^	3.80	0.5642 ± 0.0588	0.3722 ± 0.0414	0.000	1.13	0.66
Acetylcarnitine^LC^	3.82	0.8964 ± 0.1238	0.4568 ± 0.1120	0.001	1.06	0.51
5-Aminopentanoic acid^LC^	3.84	5.8135 ± 0.5834	2.9663 ± 0.4405	0.000	1.17	0.51
D-Threitol^LC^	3.84	0.7122 ± 0.0833	0.6094 ± 0.0347	0.001	1.06	0.86
3-Pyridylacetic acid^LC^	3.85	0.2082 ± 0.0183	0.1136 ± 0.0121	0.000	1.17	0.55
Deoxyguanosine^LC^	3.85	0.3297 ± 0.0975	0.1598±0.0256	0.001	1.08	0.48
2,3,4-Trihydroxybutyric acid^LC^	3.87	2.6760 ± 0.3617	1.4778 ± 0.1827	0.000	1.14	0.55
Betaine^LC^	3.87	0.4706 ± 0.0353	0.3238 ± 0.0423	0.000	1.12	0.69
D-Ribose^LC^	4.08	0.0499 ± 0.0066	0.0172 ± 0.0085	0.000	1.13	0.34
Cytosine^LC^	4.17	0.9537 ± 0.1139	0.2135 ± 0.0368	0.000	1.19	0.22
Uracil^LC^	5.08	0.1022 ± 0.0198	0.0434 ± 0.0296	0.001	1.08	0.42
Methionine^LC^	5.10	0.8950 ± 0.0830	0.3701 ± 0.0297	0.000	1.19	0.41
3-Hydroxypropionic acid^LC^	5.11	43.8395 ± 5.0814	13.3952 ± 4.8559	0.000	1.18	0.31
nicotinamide^LC^	5.11	3.7275 ± 0.4084	2.5355 ± 0.1644	0.000	1.15	0.68
Nicotinic acid^LC^	5.12	0.2603 ± 0.0304	0.1834 ± 0.0120	0.000	1.14	0.70
L-alloisoleucine^LC^	8.51	12.4753 ± 1.1999	5.3369 ± 0.3026	0.000	1.18	0.43
Methylmaleic acid^LC^	11.43	0.1281 ± 0.0141	0.0663 ± 0.0078	0.000	1.15	0.52
3-Methoxytyramine^LC^	11.43	0.0927 ± 0.0102	0.0468 ± 0.0019	0.000	1.15	0.51
Azelaic acid^LC^	17.83	1.0016 ± 0.0604	1.2211 ± 0.0702	0.003	1.01	1.22
Sebacic acid^LC^	18.46	0.3975 ± 0.0308	0.5076 ± 0.0255	0.000	1.11	1.28

^GC^represents metabolites identified with GC/TOFMS.

^LC^represents metabolites identified with LC/TOFMS.

^a^the P value was obtained with Student’s *t* test.

**Table 4:** Summary of the metabolic profiling data sets used in PCA and PLS-DA models.

Comparisons	PCA Model		PLS-DA Model		
	Number of components	R^2^X(cum)	Number of components	R^2^Y(cum)	Q^2^(cum)
All groups	6	0.893	9	0.974	0.883
Met 8 h vs Con 8 h	3	0.881	3	0.994	0.977
Met 24 h vs Con 24 h	2	0.672	2	0.985	0.933
Met 48 h vs Con 48 h	2	0.779	2	0.997	0.983
