# Supplementary material for: Metformin suppressed the proliferation of LoVo cells and induced a time-dependent metabolic and transcriptional alteration
Source: Sci Rep. 2015 Nov 30;5:17423. doi: 10.1038/srep17423 (PMC4663508; doi:10.1038/srep17423)
Supplement: Supplementary Information [file srep17423-s1.pdf]

Metformin suppressed the proliferation of LoVo cells and induced a time-dependent metabolic and transcriptional alteration

Jiaojiao He[1], Ke Wang[3], Ningning Zheng[1], Yunping Qiu[5], Guoxiang Xie[6], Mingming Su[6], Wei Jia[1,4,6], Houkai Li[1,2*]

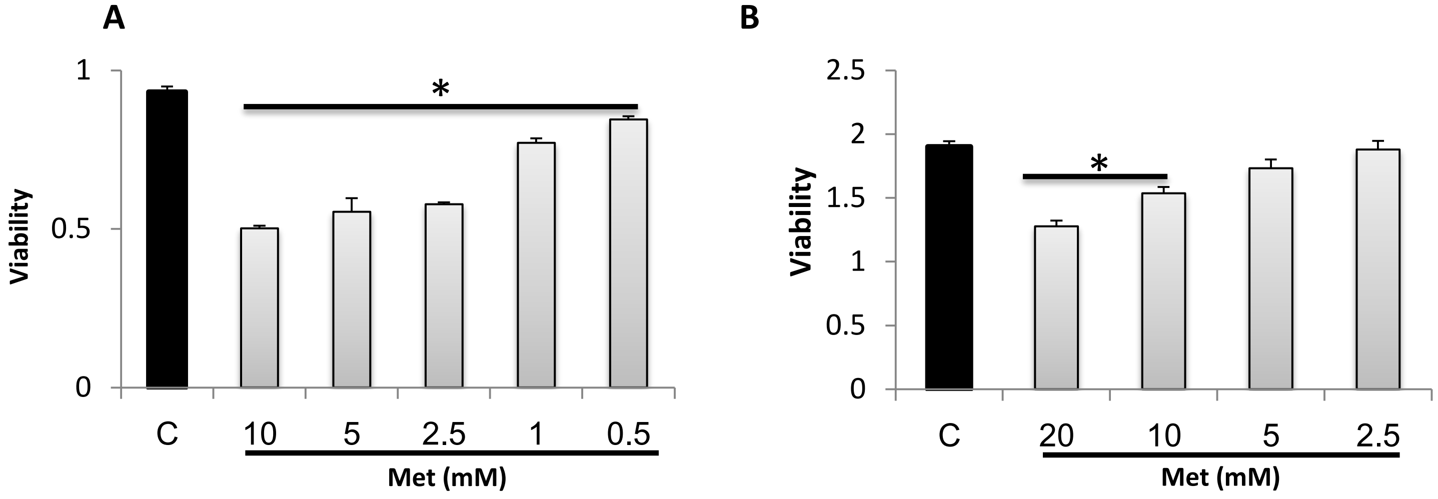

Figure S1. Metformin suppressed the proliferation of Caco-2 and HT-29 colon cancer cells. Caco-2 (**A**) and HT-29 (**B**) cells were cultured in 10% FBS DMEM and treated with or without metformin at a series of concentrations for 48h. Then, cell viability was assayed with CCK-8 according to its instruction. *indicates $P<0.05$ compared to control cells.
